# Lignocellulose utilization and bacterial communities of millet straw based mushroom (*Agaricus bisporus*) production

**DOI:** 10.1038/s41598-018-37681-6

**Published:** 2019-02-04

**Authors:** Hao-Lin Zhang, Jin-Kang Wei, Qing-Hui Wang, Rui Yang, Xiao-Jing Gao, Yu-Xi Sang, Pan-Pan Cai, Guo-Qing Zhang, Qing-Jun Chen

**Affiliations:** 1 Beijing Key Laboratory for Agricultural Application and New Technique, College of Plant Science and Technology, Beijing University of Agriculture, Beijing, 102206 China; 2 Key Laboratory of Urban Agriculture (North) of Ministry of Agriculture, College of Biological Science and Engineering, Beijing University of Agriculture, Beijing, 102206 China; 3 College of Forestry and Horticulture, Xinjiang Agricultural University, Urumqi, 830052 China; 4 Beijing Agricultural Technology Extension Station, Beijing, 100029 China; 5 Chengde Xingchunhe Agricultural Co. Ltd., Chengde, 067000 China

## Abstract

*Agaricus bisporus* is in general cultivated on wheat and rice straw in China. However, millet straw is a potential alternative resource for *Agaricus bisporus* cultivation, but this has hardly been studied. In the present study, the feasibility of millet straw based mushroom production was analyzed by three successive trials. Mature compost demonstrated high quality with total nitrogen, pH, and C/N ratio of 2.0%, 7.5, and 18:1 respectively, which was suitable for mushroom mycelia growth. During composting, 47–50% of cellulose, 63–65% of hemicellulose, and 8–17% lignin were degraded, while 22–27% of cellulose, 14–16% of hemicellulose, and 15–21% of lignin were consumed by *A. bisporus* mycelia during cultivation. The highest FPUase and CMCase were observed during mushroom flushes. Endo-xylanase had the key role in hemicellulose degradation with high enzyme activity during cultivation stages. Laccase participated in lignin degradation with the highest enzyme activity in Pinning stage followed by a sharp decline at the first flush. Yield was up to 20 kg/m^2^, as this is similar to growth on wheat straw, this shows that millet straw is an effective resource for mushroom cultivation. Actinobacteria, Bacteroidetes, Chloroflexi, Deinococcus-Thermus, Firmicutes, and Proteobacteria were the dominant phyla, based on 16S rRNA gene sequencing during composting. The key environmental factors dominating bacterial communities of the samples were determined to be pH value, cellulose content, and hemicellulose content for prewetting and premixed phase of basic mixture (P0); moisture content for phase I (PI); and nitrogen content, lignin content, and ash content for phase II (PII), respectively.

## Introduction

*Agaricus bisporus*, commonly known as button mushroom or white mushroom, is one of the most popular edible mushroom species and widely cultivated all over the world, especially in Europe, North America, China and Australasia^1,2^. In European countries, commercial production of *A. bisporus* is cultivated on a composted mixture based on wheat straw, horse or chicken manure, gypsum, and water^3,4^. The composting process involves two phases (I and II). In phase I (PI), the straw is first wettened with water and subsequently mixed with the other compounds. The stage is about 5–7 days in European countries and 15–21 day in China^4–6^. During this stage, the compost temperature increases to 80 °C due to thermophilic microorganisms. Subsequently, a pasteurization process (phase II, PII) is performed. The compost is conditioned at 45–50 °C for about 4–9 days until the ammonia level is non-toxic to *A. bisporus* mycelia, after which the temperature is reduced to about 25 °C. At the end of PII, compost can be used for (optimal) *A. bisporus* growth^4,7–9^.

As a world-wide commercial production mushroom species, *A. bisporus* can be cultivated on various composts made by different raw materials, but is always carbon and nitrogen source based. In many countries, wheat straw and horse manure are used as primary carbon and nitrogen source respectively, while chicken manure is in some cases used as an additional nitrogen source^3,4^. Nowadays, China has become the greatest producer of button mushroom with annual production in 2016 of about 3 million tons, according to the statistical data from the China Edible Fungi Association. In China, wheat and rice straw, and chicken manure are used as primary carbon and nitrogen source, respectively, while bean meal is used as an additional nitrogen source^6,10,11^.

During the composting and mushroom cultivation process, carbohydrates of plant cell walls are hydrolyzed by which monosaccharides are released which support mushroom growth^3,12^. Plant cell walls are mainly composed of cellulose, hemicellulose and lignin^13,14^. Fate of lignocellulose derived components and their degrading enzymes during composting and mushroom cultivation were reported for wheat straw based compost^1,15–17^.

Most mushroom farms in China are located in Shandong, Fujian, Gansu provinces. However, many new mushroom farms are established in the Northern part of China where rice and wheat are not widely cultivated. Local plant straws are important as an alternative carbon source to save the transport costs. Millet (*Panicum miliaceum*), is the cereal that is grown in the (semi)- arid areas of Northern China and therefore we tested in this study whether it can be used to produce compost for *A. bisporus*.

Millet just like wheat, belongs to the family Poaceae in the plant group of Monocots, and ranks sixth among the world’s most important cereal grains^18^. In China, millet is widely cultivated in arid and semi-arid regions with an annual production of about 1.78 million tons^19^. Therefore, millet straw is sufficiently available as a potential source of commercial mushroom cultivation in Northern China. However, no studies on millet based composting have currently been reported.

The aim of the present study was to determine the feasibility of mushroom cultivation using millet straw as the primary carbon source. Changes of carbohydrate compositions, lignocellulolytic enzyme levels, and bacterial diversity during composting and cultivation process were investigated.

## Results

### Physico-chemical properties of millet straw

As the first step to determine whether millet straw is suitable to create compost for *A. bisporus*, its physico-chemical properties were compared with wheat. Millet straw was collected from Inner Mongolia and wheat straw samples gathered from Xinjiang and Jiangxi of China. The data are summarized in Tables 1 and S1. Total nitrogen (w/w%) of millet straw samples was 0.55%, which is similar to that of both wheat straw samples. Moreover, ash content (w/w%) of millet straw samples was shown to be 4.50% which was about half of that of wheat straw samples. Cellulose, hemicellulose (together forming the total carbohydrate fraction), and lignin content (w/w%) of millet straw samples was 36.68%, 17.31%, and 19.38%, respectively, which were also close to those of wheat straw samples. As the composition of millet straw is similar to that of wheat and the ash content is lower, it suggests that this straw can be a good substrate to make compost.

**Table 1:** The physico-chemical properties of raw materials.

Samples	Total carbohydrates (w/w%)	Cellulose (w/w%)	Hemicellulose (w/w%)	Lignin (w/w%)	Total nitrogen (w/w%)	Ash (w/w%)	pH	Moisture (%)
Millet straw	53.98 ± 0.50	36.68 ± 0.40	17.31 ± 0.16	19.38 ± 0.21	0.55 ± 0.02	4.50 ± 0.03	5.71 ± 0.03	15.64 ± 0.69
Wheat straw-XJ	56.32 ± 0.71	36.35 ± 0.68	19.96 ± 0.06	17.19 ± 0.34	0.49 ± 0.01	8.44 ± 0.38	6.69 ± 0.14	10.36 ± 0.34
Wheat straw-JX	53.09 ± 1.47	33.98 ± 0.86	19.11 ± 0.64	21.06 ± 0.66	0.77 ± 0.00	10.22 ± 0.38	7.42 ± 0.19	15.11 ± 0.79
Chicken manure	—	—	—	—	4.40 ± 0.05	14.09 ± 0.62	5.16 ± 0.06	78.33 ± 1.15
Bean meal	—	—	—	—	7.77 ± 0.13	6.22 ± 0.10	6.43 ± 0.15	14.33 ± 0.58
Gypsum	—	—	—	—	0	100	7.02 ± 0.03	8.00 ± 0.12

Wheat straw-XJ and Wheat straw-JX were samples from Xinjiang and Jiangxi of China, respectively; ‘−’: Not detected.

### Physico-chemical properties of the compost

Three independent composting experiments were performed using millet straw as the major carbon source. Changes in compost composition during composting and mushroom cultivation were constantly monitored using onion mesh bags and basket cultivation methods^15,20^. The physico-chemical properties of compost samples during composting phases and mushroom cultivation stages of these three experiments are shown in Table 2. This includes total carbohydrate and lignin, total nitrogen, ash, content C/N ratio, pH, and moisture content (w/w%). During composting in the 3 experiment, total carbohydrate level declined from 43–45% to 27–32%, while lignin and total nitrogen increased from 20% to 25–27% and 1.2–1.4% to 1.8–2.2%, respectively. Mushroom cultivation can be divided in 5 stages (see materials and methods). During each stage samples were taken. The levels of the three components only changed slightly during each stage, and reached the final values of about 15–21% (total carbohydrate), 23–25% (lignin), and 2.1–2.3% (nitrogen), respectively after 3rd flush. To determine the C/N ratio, Carbon content was measured by the dry ashing method^21^. At the end of composting PII the C/N ratio had decreased from 33–37 to 18–19. During the composting stages, ash content continuously increased finally reaching 26–29%, while the final ash content and C/N ratio after mushroom cultivation was 32–39% and 15–18, respectively. The pH value was 7.3–7.7 at the end of PII and underwent a mild continuous decline during mushroom cultivation resulting in pH 6.2–6.4 after the 3rd flush. Moisture content at end of P0, PII, and 3rd flush were 74–77%, 68–71%, and 58–63%, respectively.

**Table 2:** Total carbohydrates, lignin, total nitrogen, ash, C/N ratio, pH, and moisture during composting and mushroom cultivation.

Phase		Total carbohydrates (w/w%)	Lignin (w/w%)	Total nitrogen (w/w%)	Ash (w/w%)	C/N ratio	pH	Moisture (%)
P0	T1	45.04 ± 0.75	20.03 ± 0.09	1.22 ± 0.01	18.17 ± 0.29	37.26	7.72 ± 0.10	74.12 ± 0.23
	T2	43.49 ± 1.69	20.40 ± 0.52	1.42 ± 0.04	14.50 ± 0.00	33.45	6.79 ± 0.15	77.34 ± 0.04
	T3	42.80 ± 2.15	20.25 ± 0.85	1.39 ± 0.02	18.67 ± 0.29	32.51	7.13 ± 0.11	73.96 ± 0.12
PI	T1	41.21 ± 0.21	21.01 ± 0.12	1.33 ± 0.08	20.67 ± 0.76	33.14	7.86 ± 0.02	73.29 ± 0.27
	T2	37.52 ± 0.65	22.42 ± 0.56	1.76 ± 0.02	25.17 ± 0.29	23.62	7.54 ± 0.04	76.79 ± 0.08
	T3	37.00 ± 0.55	25.13 ± 0.39	1.77 ± 0.02	23.50 ± 0.50	24.01	7.43 ± 0.02	73.34 ± 0.25
PII	T1	31.91 ± 0.56	25.04 ± 0.85	1.76 ± 0.01	25.83 ± 0.29	23.41	7.33 ± 0.09	67.92 ± 0.46
	T2	28.55 ± 0.79	26.42 ± 1.11	2.09 ± 0.06	29.00 ± 0.50	18.87	7.65 ± 0.04	70.96 ± 0.05
	T3	28.18 ± 0.35	27.34 ± 0.19	2.17 ± 0.01	27.83 ± 0.29	18.47	7.42 ± 0.03	67.54 ± 0.29
Filling	T1	26.97 ± 0.38	24.87 ± 0.17	2.16 ± 0.05	27.17 ± 0.29	18.73	6.62 ± 0.03	67.10 ± 0.10
	T2	23.54 ± 0.26	24.37 ± 0.15	2.21 ± 0.02	31.83 ± 0.29	17.14	6.55 ± 0.03	69.94 ± 0.47
	T3	22.85 ± 0.09	25.95 ± 0.11	2.37 ± 0.06	30.00 ± 0.87	16.41	6.46 ± 0.01	67.45 ± 0.25
Pinning	T1	26.38 ± 0.53	21.02 ± 0.49	2.20 ± 0.04	28.50 ± 0.00	18.05	6.55 ± 0.02	65.93 ± 0.12
	T2	24.23 ± 0.35	21.35 ± 0.11	2.23 ± 0.03	32.17 ± 0.29	16.90	6.53 ± 0.03	66.32 ± 0.41
	T3	24.43 ± 0.44	22.47 ± 0.27	2.33 ± 0.03	30.83 ± 0.58	16.49	6.39 ± 0.02	66.03 ± 0.29
1^st^ flush	T1	21.44 ± 0.18	23.43 ± 0.04	2.19 ± 0.14	32.00 ± 0.50	17.25	6.22 ± 0.01	58.55 ± 0.45
	T2	16.79 ± 0.36	23.48 ± 0.14	2.34 ± 0.05	35.67 ± 0.29	15.27	6.43 ± 0.03	62.32 ± 0.48
	T3	17.87 ± 0.13	24.45 ± 0.21	2.32 ± 0.03	33.83 ± 0.58	15.84	6.19 ± 0.04	63.10 ± 0.26
2^nd^ flush	T1	19.65 ± 0.28	23.65 ± 0.40	2.14 ± 0.03	33.17 ± 0.29	17.35	6.38 ± 0.04	58.15 ± 0.82
	T2	16.41 ± 0.50	24.40 ± 0.49	2.32 ± 0.09	36.50 ± 0.50	15.21	6.35 ± 0.05	60.37 ± 0.12
	T3	16.66 ± 0.36	24.75 ± 0.10	2.24 ± 0.09	34.83 ± 0.29	16.16	6.26 ± 0.04	63.38 ± 0.23
3^rd^ flush	T1	21.00 ± 0.04	23.26 ± 0.36	2.13 ± 0.01	32.33 ± 0.29	17.65	6.35 ± 0.02	58.18 ± 0.09
	T2	15.08 ± 0.10	23.90 ± 0.81	2.28 ± 0.02	38.67 ± 0.29	14.94	6.19 ± 0.04	62.65 ± 0.52
	T3	18.57 ± 0.03	24.56 ± 0.02	2.27 ± 0.01	34.00 ± 0.50	16.15	6.43 ± 0.11	60.38 ± 0.88

P0, PI, and PII: end of premix, phase I, and phase II, respectively; T1, T2, T3: different trials; Results represent mean ± standard deviation (n = 3).

### Absolute changes of total carbohydrate and lignin during composting and cultivation level

**Figure 1:**
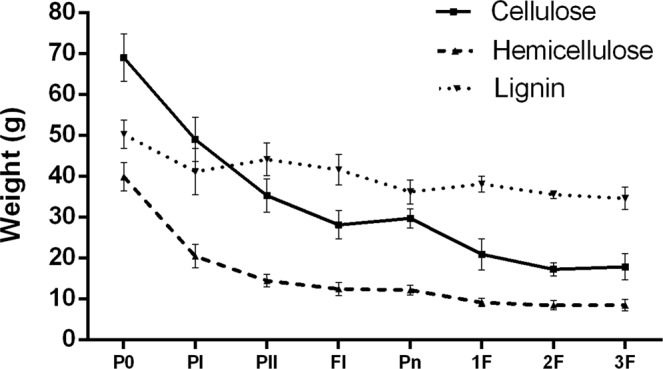
The lignocellulose contents of compost in different phases. P0, PI, and PII: end of premix, phase I, and phase II, respectively.

In the former paragraph we described the relative changes in total carbohydrate and lignin levels. To obtain insight in the absolute changes we determined the fresh and dry weight of the compost samples and calculated the absolute changes using the data from Table 2. Changes of cellulose, hemicellulose, and lignin contents were visualized in Fig. 1 and Table 3. At the end of PII, 32–33% of the dry matter was consumed, which included the loss (consumption) of cellulose, hemicellulose, and lignin with 47–50%, 63–65%, and 8–17%, respectively. During mushroom cultivation, 6–12% of dry matter, 22–27% of cellulose, 14–16% of hemicellulose, and 15–21% of lignin were most likely primarily consumed by *A. bisporus*. So, the total loss from P0 till the cultivation is, 38–46% of dry matter finally including about 73–77% of cellulose, 78–81% of hemicellulose, and 28–37% of lignin (Table 3).

**Table 3:** The mass balance, dry matter, cellulose, hemicellulose and lignin of compost.

Phase		Fresh compost		Dry matter		Cellulose		Hemicellulose		Lignin	
		Weight/g	Loss rate/%	Weight/g	Loss rate/%	Weight /g	Loss rate /%	Weight/g	Loss rate/%	Weight/g	Loss rate/%
P0	T1	1000.00	—	258.80	—	73.54	—	43.01	—	51.84	—
	T2	1000.00	—	226.60	—	62.46	—	36.10	—	46.23	—
	T3	1000.00	—	260.40	—	70.97	—	40.48	—	52.73	—
PI	T1	704.00	29.60	188.04	27.34	54.22	26.28	23.28	45.87	39.51	23.79
	T2	700.80	29.92	162.66	28.22	43.47	30.40	17.56	51.35	36.47	21.11
	T3	707.20	29.28	188.54	27.60	49.20	30.67	20.56	49.22	47.38	10.15
PII	T1	537.72	16.63	172.50	6.00	39.27	20.33	15.78	17.44	43.19	−7.11
	T2	529.45	17.13	153.75	3.93	31.15	19.73	12.75	13.33	40.62	−8.99
	T3	547.23	16.00	177.63	4.19	35.24	19.68	14.82	14.16	48.56	−2.25
Filling	T1	519.16	1.86	170.80	0.65	31.90	10.01	14.16	3.77	42.48	1.38
	T2	511.24	1.82	153.68	0.03	25.12	9.65	11.05	4.70	37.45	6.86
	T3	530.43	1.68	172.66	1.91	27.34	11.13	12.11	6.69	44.80	7.13
3^rd^ flush	T1	335.53	18.36	140.32	11.78	19.90	16.32	9.57	10.67	32.64	18.98
	T2	373.96	13.63	140.05	6.02	14.24	17.42	6.87	11.57	33.47	8.61
	T3	387.11	14.33	153.37	7.40	19.45	11.11	9.03	7.63	37.67	13.53

P0, PI, and PII: end of premix, phase I, and phase II, respectively; T1, T2, T3: different trials.

### Lignocellulosic enzyme activities during composting and cultivation of *A bisporus*

**Figure 2:**
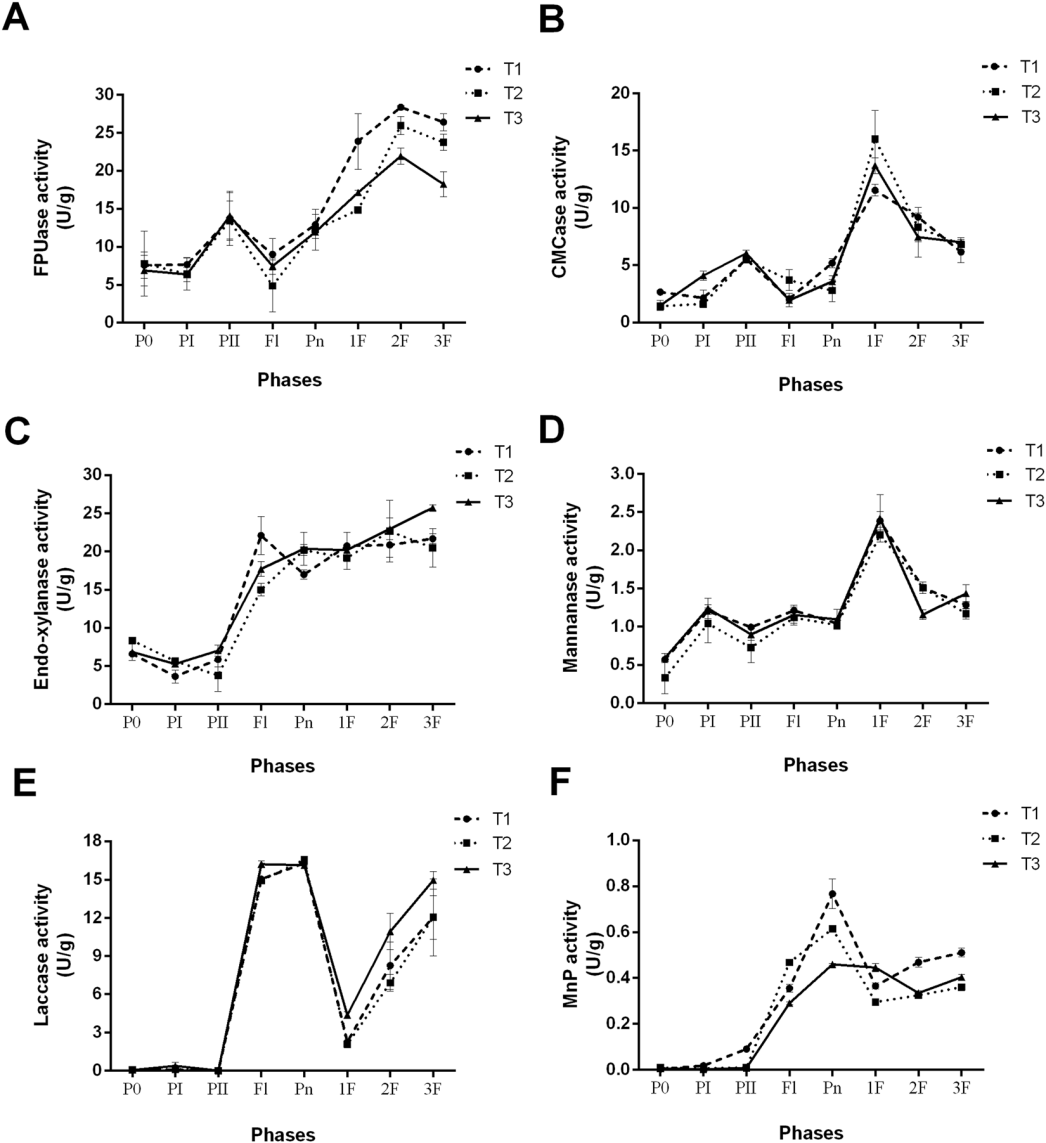
Lignocellulosic enzyme activities in various stages. (**A**) FPUase; (**B**) CMCase; (**C**) endo-xylanase; (**D**) mannanase; (**E**) laccase; (**F**) MnP; P0: end of premix; PI: end of phase I; PII: end of phase II; Fl: Filling; Pn: Pinning; 1F: end of 1^st^ flush; 2F: end of 2^nd^ flush; 3F: end of 3^rd^ flush.

The lignocellulose compounds are degraded by enzymes made by the microbiome in the compost as well as *A. bisporus*. We tested the activity of several enzymes during the composting processes well as during cultivation of the mushrooms. We selected the following enzymes because they are known to degrade one of the lignocellulose compounds. Further it is known the *A. bisporus* can make these enzymes. Whether the microbiome responsible for the composting makes similar enzymes will be tested. The enzymes include filter paper cellulase (FPUase, A), carboxymethyl cellulase (CMCase, B) that both can degrade cellulose, endo-xylanase (C) and mannanase (D) can degrade hemicellulose, and laccase (E) and manganese peroxidase (MnP, F) can degrade lignin. The data are shown in Fig. 2. Cellulose and hemicellulose are fastest during the composting process and their degradation continues during the cultivation of the mushrooms (Fig. 1). This is in contrast with the measured enzyme activities. The enzymes degrading cellulose (Fig. 2A,B) as well as those degrading hemicellulose (Fig. 2C,D) have the highest activity during mushroom cultivation and have a markedly lower activity level during composting. This suggests that in addition to these 4 enzymes the microbiome involved in composting secretes additional enzymes that can degrade these carbohydrates. During both composting and mushroom cultivation lignin is degraded at a markedly lower speed than the 2 carbohydrates Fig. 1. The 2 lignin degrading enzymes are made at a high level during mushroom cultivation but are almost not active during the composting. Therefore, it seems probable that also in this case the microbiome uses other enzymes for the degradation of lignin.

### Bacterial communities during composting

**Figure 3:**
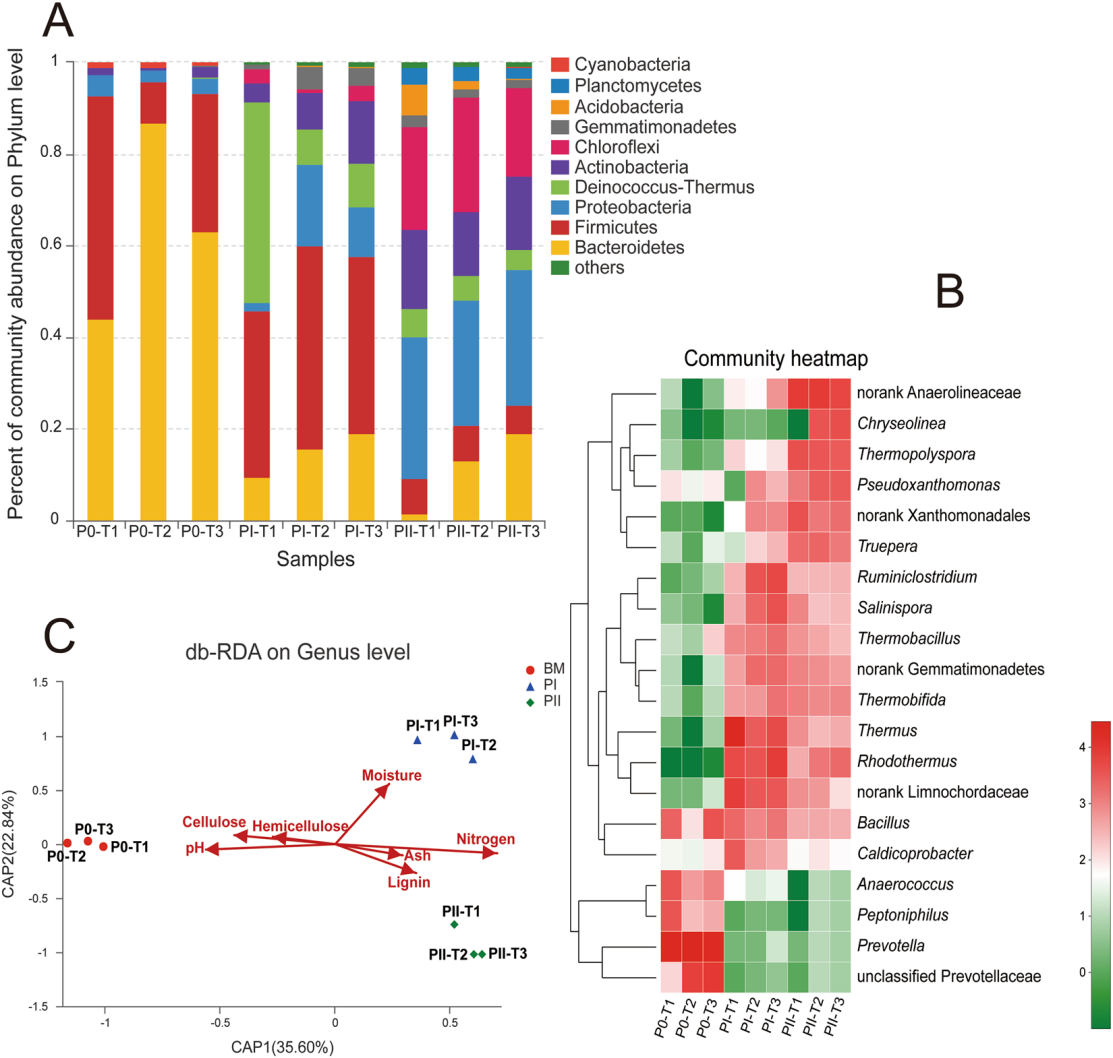
Bacterial community analysis during composting. (**A**) Relative abundance of bacterial communities (phylum level). (**B**) Heat map analysis (genus level). (**C**) db-RDA analysis between environmental factors and bacterial groups (OTU level). OTU: operational taxonomic units; P0: end of premix; PI: end of phase I; PII: end of phase II; T1, T2, T3: different trials. The heatmap plot indicates the relative abundance of genera in different samples. The phylogenetic tree was calculated using the neighbour-joining method. The color intensity is proportional to the relative abundance of bacterial genera.

Bacterial communities during composting were determined by high throughput sequencing based on V3-V4 region of 16S rRNA genes. The sequence information and calculated microbial diversity index are summarized in Table 4. The number of sequences per sample ranged from 44010 to 57742, and the OTUs of each sample ranged from 323 to 567. In mature compost samples of T2 and T3, the bacterial taxa represented 17 phyla, 37 classes, and 85 orders. The composition of the bacterial communities at different stages (P0, PI, and PII) demonstrated substantial differences, while those in the same stage, but from different trials were rather similar (Fig. 3A). Bacteroidetes, Firmicutes, and Proteobacteria were the dominant phyla in P0 samples, and *Prevotella* was the dominant genus in all the three trials (Fig. 3B). Firmicutes, Bacteroidetes, Proteobacteria, Deinococcus-Thermus, and Actinobacteria were the dominant phyla in PI (Fig. 3A). Proteobacteria, Chloroflexi, Actinobacteria, and Firmicutes were the most abundant bacteria taxa in PII (Fig. 3B). The db-RDA analysis on genus level between the environmental factors and bacterial communities was visualized in Fig. 3C. Samples in different stages were separated into three clusters of P0, PI, and PII, respectively. The key environmental factors dominating bacterial communities of the samples were determined to be pH value, cellulose content, and hemicellulose content for P0; moisture content for PI; and nitrogen content, lignin content, and ash content for PII, respectively.

**Table 4:** Summary statistics of Illumina MiSeq PE300 sequencing bacterial 16S rDNA regions of composting samples.

Phase		Sequence number	Phylum	Class	Order	Family	Genus	Species	OTU	Chao	Shannon
P0	T1	57742	13	25	59	111	247	424	424	498.20	3.00
	T2	44010	9	21	43	82	187	264	323	362.82	1.58
	T3	51019	13	25	61	116	251	344	437	530.38	2.83
PI	T1	55165	13	31	77	138	226	310	413	476.63	2.83
	T2	45220	15	35	87	155	259	356	503	566.38	4.38
	T3	55362	16	39	95	173	295	396	567	636.84	4.12
PII	T1	49786	16	36	81	138	202	272	378	449.38	3.75
	T2	49841	17	37	85	153	232	310	457	498.36	4.12
	T3	46388	17	37	85	150	228	311	444	515.51	3.91

Note: P0, PI, and PII: end of premix, phase I, and phase II, respectively; T1, T2, T3: different trials; BE: biological efficiency; OTU: operational taxonomic units; Chao: Chao index, estimating the species abundance of the community; Shannon: Shannon index, calculating community diversity.

### Yield and biological efficiency (BE)

The mushroom yield of the three trials was 22.10, 21.30, and 24.47 kg/m^2^ respectively without any supplements (Table 5). For each trial, the yield of 1^st^ flush accounted for the highest proportion among the total, followed by 2^nd^ flush. BE of the three trials was 68.4%, 70.42%, and 72.58%, respectively.

**Table 5:** The yield and biological efficiency of mushroom.

Trial	Yield (kg/m^2^)			Percentage (%)			Total yield (kg/m^2^)	BE (%)
	1^st^ flush	2^nd^ flush	3^rd^ flush	1^st^ flush	2^nd^ flush	3^rd^ flush		
T1	10.22 ± 0.38	9.27 ± 0.25	2.60 ± 0.26	46.26	41.97	11.77	22.10	68.40
T2	12.46 ± 0.25	7.58 ± 0.20	1.26 ± 0.13	58.48	35.59	5.93	21.30	70.42
T3	10.32 ± 0.38	8.82 ± 0.36	5.33 ± 0.23	42.16	36.06	21.78	24.47	72.58

T1, T2, T3: different trials; BE: biological efficiency.

### Scanning electron microscope analysis

**Figure 4:**
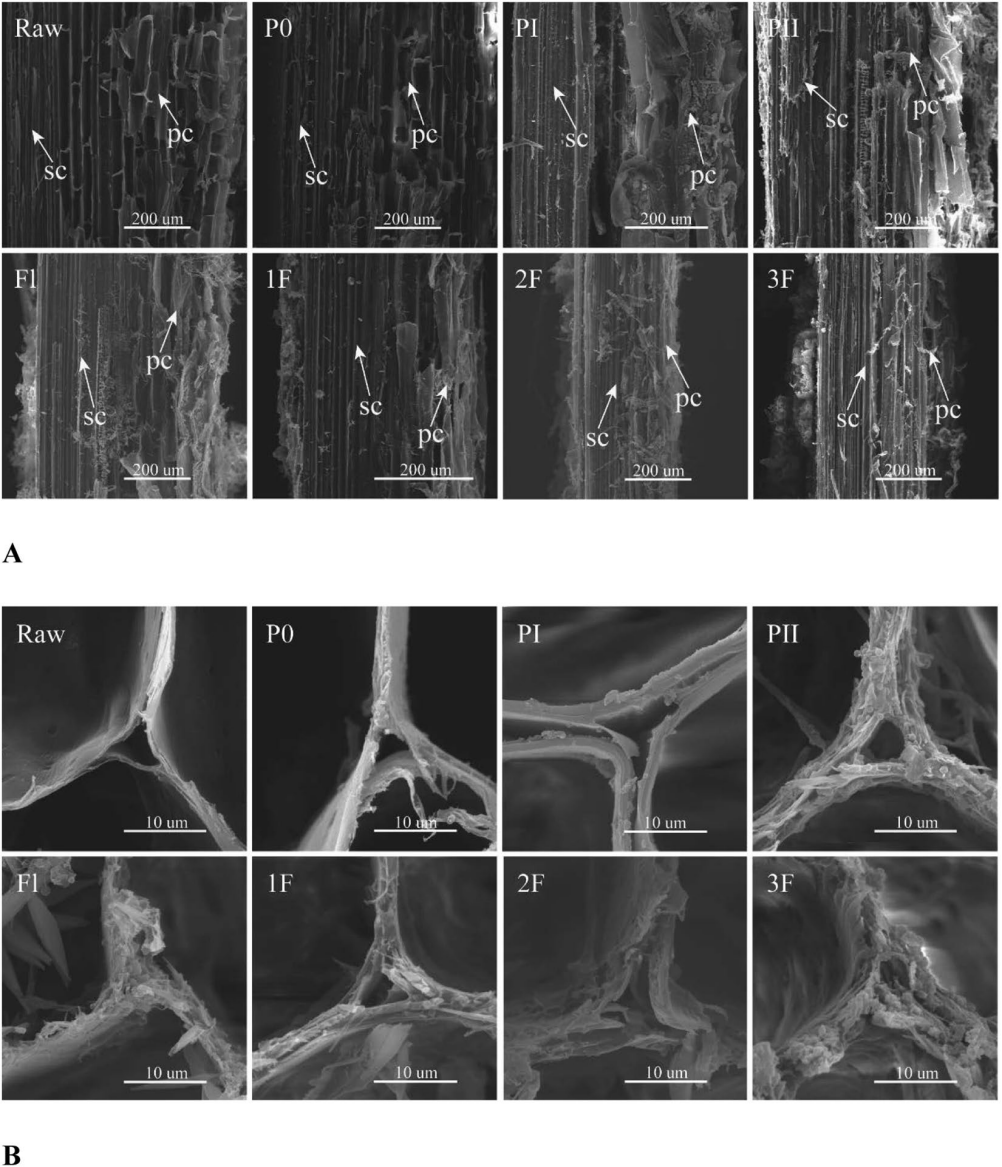
Scanning electron microscope analysis of millet straw in various stages. (**A**) Vertical section of straw; (**B**) transverse section of parenchyma cells; Raw: raw materials; P0: end of premix; PI: end of phase I; PII: end of phase II; Fl: Filling; Pn: Pinning; 1F: end of 1^st^ flush; 2F: end of 2^nd^ flush; 3F: end of 3^rd^ flush; sc: sclerenchymatous cells; pc: parenchyma cell.

Microstructural changes of millet straw during composting and mushroom cultivation are shown in Fig. 4. Longitudinal sections show that, cell wall structures were intact in raw materials (Raw) and P0 stage, but continuously degraded from PI to 3F stage (Fig. 4A). The sclerenchymatous cells (sc, mainly containing lignin) and the parenchyma cells (pc, mainly containing cellulose and hemicellulose) can be easily seen in of the millet straw raw material. Many needle-like crystals were found on the millet straw surfaces at Fl stage (Fig. 4A-Fl). Cellular frame structure of parenchyma cells became gradually blurred from PI on, while sclerenchymatous cell structures were still very clear. These results corresponded to the changes of the levels of cellulose, hemicellulose, and lignin as summarized in Table 3. Transverse section show that the cell wall of parenchyma cells became continuously loosened (Fig. 4B). They were digested by composting microorganisms and mushroom mycelia.

## Discussion

In the present study, we focused on the feasibility of mushroom cultivation using millet straw as the primary carbon source. The mushroom yield of millet straw based compost was over 20 kg/m^2^ and in these cases no supplements were added. Previous studies showed that total yield of three flushes is about 25–35 kg/m^2^ when supplements are used^22–25^. The supplementation can accurately equilibrate the nitrogen content and the C/N ratio of compost, and increase production yields by up to 5–20%^26–28^. BE of our study was 68.40%, 70.42%, and 72.58%, respectively. BE of T1 was the lowest since the physico-chemical properties of compost at the end of PII were out of suggested ranges with 23% of C/N ratio and 1.76% of nitrogen content^17,24,29^. In previous study of wheat straw and chicken manure based compost, mushroom yield and BE were 18.90 kg/m^2^ and 88.7.9%, respectively^26^. It suggests that millet straw based compost is an effective cultivation medium for the button mushroom.

In European countries, wheat straw is preferred for mushroom compost since it is firm, does not lose its texture easily, and is available in large quantities. Moreover, barley, oat, and rye straw can also be used successfully. Of these three, rye straw most resembles wheat straw, while the other two are softer, absorb water readily, and lose their texture more quickly^30^. In China, rice and corn straw are also used successfully. Rice straw is closer to wheat straw, while corn straw more resembles barley or oat straw and loses its texture more quickly^6,31,32^. Up till now, few studies on millet straw have been reported for both its physico-chemical properties and application on mushroom cultivation.

Mushroom compost production is a process (divided into two stages PI and PII) involving the bioconversion of raw materials into a substrate supporting the growth of *A. bisporus*. In the present study, a 17-day PI stage was performed, which is about 2–3 times longer than that of European countries^4–6^. PI is characterized by high temperatures up to 80 °C. Thermophilic microbiota proliferate and degrade the carbohydrates and proteins, which results in the release of heat and ammonia. These reactions cause the raw materials (e.g. wheat straw and rice straw) to soften^15,24^. Since millet straw is thicker and longer than wheat straw, it is harder to be degraded. In our pre-experiments, a longer PI stage about 16–18 d can help the compost production reach the quality requirement for mushroom cultivation.

In the present study, we found that total carbohydrates and nitrogen of millet straw were close to not only that of wheat straw from Xinjiang and Jiangxi, but also those of wheat straw used in the Netherlands^3^. Moreover, contents of cellulose, hemicellulose, and lignin of millet straw were very close to those of wheat straw from Xinjiang and Jiangxi (Table 1). On the other hand, lignin content of millet straw (19.38%) was lower than that of wheat straw from Netherlands (26–27%)^3,15^. Based on present and previous studies, less than half of the total lignin was consumed during composting and mushroom cultivation^16^. Therefore, the difference of lignin of millet straw and wheat straw can not remarkably affect the mushroom yield. It suggests that millet straw demonstrates similar physicochemical properties with wheat straw, and can be used as high-quality raw materials for mushroom cultivation.

Since mushrooms consist of more than 92% water, moisture content is one of the key factors for mushroom cultivation. Water-holding capacity of the main raw materials is a very important parameter for both composting and mushroom cultivation^30^. In the present study, moisture content of the compost underwent a continuous decline from about 75% at the beginning to 60% at the 3^rd^ flush, falling well in the recommended moisture variation range described by van Griensven^30^ of 75%, 72%, 67%, and 60% at the end of P0, PI, PII, and growing. Millet straw demonstrates similar water-holding capacity as wheat straw during the whole mushroom production process.

In the present study, total nitrogen and C/N ratio of the three trials were designed to be about 1.2–1.4% and 33:1–37:1, respectively, which is required for high quality compost^30,33^. At the end of PII, the physico-chemical properties of compost were the key factors to determine the mushroom yield, which need to be within specific ranges to obtain maximum mushroom yield^33^. The moisture, total nitrogen, pH, and C/N ratio should be in the range of 68–72%, 2.0–2.4%, 7.4–7.6, and 17–20:1, respectively^17,24,29^. After composting, total nitrogen, pH, and C/N ratio of T2 and T3 samples were about 2.1–2.2%, 7.4–7.7, and 18:1–19:1, respectively, suggesting that the two compost samples based on millet straw were of high quality and suitable for mushroom growth. At the P0 stage, T1 sample possessed high pH of 7.72 and C/N ration of 37:1, but low total nitrogen of 1.22%. After composting, the T1 sample was still not very well with high C/N ration of 23% and low total nitrogen of 1.76%. Since the millet straw was purchased from different local farmers of Inner Mongolia, the uniformity was not very high.

Lignin is a complex cross-linked phenolic polymer, particularly important in the formation of plant cell walls, and hard to be degraded^34,35^. In the present study, 8–17% and 15–21% of lignin was consumed during composting and mushroom cultivation, respectively. In contrast, about 3% and 15% of total lignin was respectively degraded during composting and mushroom mycelia growth in the wheat straw in previous study^15^. Wei *et al*.’s^36^ study bases on different types of straw composting from wheat, rice, corn and soybean revealed that the degrading ratio of lignin was continuously increased during composting. About 8–20% of total lignin of the assayed straw groups was degraded at the 20th day. In our study, a longer PI stage of about 2–3 times the length used in European studies was used to obtain high quality compost products. After the longer stage of PI, more lignin was degraded comparing with other studies.

In our study, 47–50% of cellulose and 63–65% of hemicellulose was degraded by composting microorganisms at the end of PII, while 50% of both cellulose and hemicellulose were metabolized in Jurak *et al*.’s report on wheat straw based compost^15^. On the other hand, very low laccase and MnP activities were detected at the end of PI and PII. This might be caused because most of the dominant microbes are thermophilic and function during the high temperature stages. Based on the results of the high throughput sequencing, microorganisms from genera *Thermobifida*, *Rhodothermus*, and *Thermus* were the dominant species during composting (Fig. 3). They have been reported to participate in lignin degradation^37–39^. Szekely, *et al*.^40^ reported that *Thermus thermophilus* was the dominant species during PI, while species of genera *Thermobifida* and *Pseudoxanthomonas* participated in the degradation of lignocellulose. It can also explain why about half of the cellulose and hemicellulose were consumed while all the tested lignocellulolytic enzyme activities were very low during composting especially during at PI stage.

Lignocellulolytic enzymes of composting microorganisms and mushroom *A. bisporus* play a key role in lignocellulosic utilization during the whole mushroom production. In the present study, cellulose was hydrolyzed by both FPUase and CMCase. The highest activities of the two enzymes during composting stages were observed at the end of PII. On the other hand, the highest degradation rates of cellulose (about 26–31%) happened during PI. This might be because the duration of PI was 17 d, and most cellulases during PI were thermophilic. The highest CMCase activity during mushroom cultivation detected at 1F was 11.5–16.0 U/g. Previous studies on wheat straw based composting revealed that CMCase activity at spawing, induction, 1F, 2F, and 3F phases were 0.6, 1.4, 13.0, 11.7, and 6.6 U/g respectively, which variation in time is similar to our results^28^. A similar trend was also observed by Jurak, *et al*.^16^. The CMCase activity increased from Fl to 1F, and underwent a slight decrease in 2F.

Degradation of xylan (the major component of hemicellulose) is a very complex process promoted by many enzymes called xylanolytic enzyme system, including endo-xylanase (endo-1, 4-β-xylanase, E.C.3.2.1.8), β-xylosidase (xylan-1, 4-β-xylosidase, E.C.3.2.1.37), α-glucuronidase (α-glucosiduronase, E.C.3.2.1.139), etc. Among them, endo-xylanases are key enzymes responsible for the hydrolysis of hemicellulose and act on the homopolymeric backbone of 1, 4-linked β-D-xylopyranose by which xylooligomers (endo-) are produced^41,42^. Endo-xylanase activity continuously increased during the mycelia growth and maintained high level of about 20 U/g during mushroom flushes. Arce-Cervantes, *et al*.^28^ also reported that the greatest endo-xylanase activity occurred at the end of 1^st^ break in unsupplemented wheat straw based compost. This might be caused because pentose produced by endo-xylanases might provide nutrition for mushroom mycelia growth.

In the present study, Laccase activity reached the highest level during mushroom mycelia growth but significantly declined during the first mushroom flush. A previous study also suggested that multicopper enzyme and MnP were very important in *Agaricus* spp. during mycelia growth^43^. Another study also showed that the level of laccase activity was greatest at the initial stage of cultivation, lowest at 1st flush, and continuous increased during three flushes^28^. It is rather similar to our study. Laccase secreted by mushroom mycelia participates in metabolic processes before fruiting bodies appear^44^. That is why the levels of laccase activity declined rapidly after vegetative mycelia growth.

The cell walls of straw of grasses are composed of lignin, cellulose, and hemicellulose^45^. Microstructure of millet straw during mushroom production was visualized using SEM. Just like our observation, a previous SEM study on wheat straw based mushroom composing also revealed that many of the plant fibers became separated, but the final material still retained considerable structural integrity^46^. Needle-like crystals found on the millet straw surfaces at Fl stage were speculate to be calcium oxalate which was produced by mushroom mycelia and similar to earlier research on wheat straw composing^46,47^. The crystals can reduce calcium ion concentrations and improve adaptability of mushroom mycelia to the environment.

Based on 16S rRNA sequencing, 17 phyla, 37 classes, and 85 orders were obtained in the mature compost samples, which was similar to a previous study on PII which showed that 16 phyla and approximate 80 orders were present^48^. It’s worth mentioning that the number of bacterial communities detected by high-throughput sequencing were higher than those using DGGE, RFLP, and clone library sequencing methods^6,40,49,50^. Based on the Chao and Shannon indexes, Samples of T2 and T3 in PI possessed the highest bacterial community diversity. Bacterial community diversity of T1 samples in different stages presented highest level at PII and lowest level at PI. Previous literature also reveals that microbial communities continuously change to adapt to the nutrient and environmental changes during composting^5,51^.

In the present study, *Prevotella* was the dominant genus in P0 samples. *Prevotella* spp. most likely came from chicken manure since they are widely distributed in animal intestines^52,53^. The abundance of *Prevotella* spp. underwent a significant decline during PI, which might be caused by the high temperature at this stage. During PI, microorganisms from genera *Ruminiclostridium*, *Bacillus*, *Thermobacillus*, *Lactobacillus*, and *Caldicoprobacter* of phylum Firmicutes, genus *Thermus* of phylum Deinococcus-Thermus, genera *Bacteroides* and *Rhodothermus* from of phylum Bacteroidetes, and genera *Thermobifida* and *Salinispora* of phylum Actinobacteria were dominant species, many of which were thermophilic and relative to cellulose degradation (Fig. 3B). Previous studies on 6 days’ PI demonstrated that Firmicutes, Proteobacteria, Actinobacteria, and Bacteroidetes were most abundant phyla^48^. Partanen, *et al*.^54^ reported that bacteria from the phyla Actinobacteria, Bacteroidetes, Firmicutes, Proteobacteria and Deinococcus-Thermus were enriched at different stages of the mushroom composting process. Bacteria from phylum Deinococcus-Thermus are known for their resistance to high temperature, and take part in lignocellulose degradation at high temperature.

Species from genus *Anaerolineaceae* of phylum Chloroflexi, genus *Thermopolyspora* of phylum Actinobacteria, genera *Pseudoxanthomonas* and *Xanthomonadales* of phylum Proteobacteria, and genus *Truepera* of phylum Dienococcus-Thermus were enriched in PII. A previous study using high-throughput sequencing declared that Firmicutes, Proteobacteria, and Actinobacteria were dominant phyla^48^. Szekely, *et al*.^40^ using DGGE and T-RFLP methods to analyze bacterial succession during mushroom composting, revealed that *Pseudoxanthomonas*, *Thermobifida*, and *Thermomonospora* species were the dominant genera in mature compost. They were supposedly related to cellulose-degradation. Zhang, *et al*.^5^ also reported that cellulolytic actinomycetal species from genus *Thermopolyspora* were highly enriched in the wheat straw based mature mushroom compost. In the current study, bacterial communities and dominant taxa of millet straw based mature compost were similar to those of wheat straw based ones, suggesting that it is feasible to produce high quality mushroom using millet straw as the primary carbon source.

Up to now, few studies reported on relations of environmental factors and bacterial communities. In the present study, pH of T1 samples at P0 was significantly higher than that of T2 and T3 (Table 2), which might cause the differences in physico-chemical properties, lignocellulose components and enzymes, bacterial communities, and mushroom yield. Ross and Harris^55^ declared that yield and quality of *Agaricus* mushrooms were dependent on the presence of thermophilic microorganisms during composting. A previous study based on yield revealed that moisture content during composting and aeration of the compost heap were significant factors during composting^56^. Microbial communities of compost interacted with the nutrient and environmental elements^5,51^. Many studies reported that nitrogen rich supplementation during PII can increase mushroom yield, indicated that nitrogen content is one of the key factors in the stage^24,26,27,57^.

In summary, millet straw based compost is suitable for *A. bisporus* mushroom cultivation. After 17-day PI and 9-day PII, physico-chemical properties of compost were as good as those of wheat straw based compost applied in factory production. Lignin was partially degraded during PI while in other studies this is not the case. This degradation was achieved because the duration of this stage was much longer than that used in European countries. FPUase, CMCase, endo-xylanase, mannanase, laccase, and MnP participated in lignocellulose degradation. Actinobacteria, Bacteroidetes, Chloroflexi, Deinococcus-Thermus, Firmicutes, and Proteobacteria were the dominant phyla during composting, while pH value, moisture content, and nitrogen content were deduced to be key environmental factors in P0, PI, and PII stages, respectively.

## Materials

### Composting and cultivation

Composting and mushroom cultivation were performed in Chengde Xingchunhe Agricultural Co. Ltd., Chengde, Hebei province, China. Millet straw was purchased from Chifeng, Inner Mongolia, China. Other raw materials were purchased from local markets. The compost formula was determined based on the commercial mushroom compost with the initial nitrogen content of 1.6% and as follow: millet straw (43 t), chicken manure (45 t), bean meal (3.6 t) and gypsum (4 t). The composting process followed the modified standards of the Netherlands including phase 0 compost (P0, including pre-wetting for 3 days and premix of basic mixture), phase I compost (PI, 17 days), and phase II compost (PII, 9 days)^1,28,30^. A turning of the compost will happen in the first fermentation tunnel (23 × 5.5 × 4.5 m) when the temperature rises up to 80 °C during PI. In the present study, four turning was done which was about every three or four days. The cultivation process included Filling (Fl, 19 days), Pinning (Pn, about 14 days), 1^st^ flush (1F, about 4 days), 2^nd^ flush (2F, about 7 days), and 3^rd^ flush (3F, about 7 days). Commercial *A. bisporus* strain A15 (Sylvan, USA) was used in this study with the inoculation quantity of 0.5%. Commercial peat purchased from Jilin Province, Northeast China was used as the casing materials. The casing used should be with the humidity of 75% and the thickness of 35–40 mm. The casing formula was as follow: the fresh peat (1 m^3^), calcium carbonate (20 kg), lime (15 kg), water (25–28 L), and pH 7.8. Mushrooms were harvested every day at their optimal commercial development stage during three flushes. Yield and biological efficiency (BE) were subsequently calculated by mushroom kilograms per cultivated area and total fresh mushroom weight divided by initial total substrate dry weight, respectively^26^.

### Sampling

In the present study, three successive trials (T1, T2, and T3) were conducted. During sampling, the onion mesh bag method as Jurak, *et al*.^15^ described was used. At the end of premix, samples from 10 random points (3.0 kg each) were taken and thoroughly mixed (named as P0 samples). Subsequently, 25.0 kg of the P0 samples were divided into five onion mesh bags (5.0 kg each) and randomly placed at 50 cm below the compost surface in the first fermentation tunnel. When turning happened, the compost in each bag was respectively mixed and transferred into another new bag. Another P0 samples (1.0 kg) from the rest were divided into equal division (50.0 g each) for laboratory studies including physical-chemical properties, enzymatic activities, electron microscope observation, and microbial diversity. At the end of PI and PII, compost in each bag was weighed, thoroughly mixed, and sampled (1.0 kg). After sampling, the rest PI samples were equally reconfigured into 5 bags and placed in the second fermentation tunnel as in the first one described above (27 × 5 × 4.5 m). During mushroom cultivation, both layer and basket cultivation were used in the standard mushroom houses. Mature compost with spawn was taken into polyethylene boxes (45 × 32 × 23 cm). Thirty boxes were used, and each of them contained 15.0 kg of the substrate^20,25^. At the end of Fl, Pn, 1F, 2F, and 3F, five boxes were randomly selected and weighted^1,16^. Substrate (200 g) at about 10 cm below the surface was taken from each box, thoroughly mixed, and storage for further laboratory studies.

### Determination of physico-chemical properties

Raw materials and samples were analyzed for their pH values and contents of moisture, nitrogen, carbon, ash, cellulose, hemicellulose, and lignin. The pH was analyzed in a 1:10 (w/v) fresh substrate water extract using a pH meter. Moisture content was assayed using fresh samples by the dry weighing method^17^. Frozen fresh samples were freeze dried and milled (<1 mm) for determination of other contents^3,16^. Total nitrogen was determined using the modified Kjeldahl method^58,59^. Ash and carbon content were assayed using the dry ashing method^21^. The C/N ratio was obtained based on total nitrogen of the Kjeldahl method and carbon content of the dry ashing method^21,29^. Cellulose and hemicellulose contents were determined by High Performance Liquid Chromatography (HPLC) according to the laboratory analytical procedure (LAP) of the National Renewable Energy Laboratory (NREL) (version 08-03-2012)^16,60–62^. In brief, dry samples (0.5 g) were treated with 72% (w/w%) H_2_SO_4_ (3.0 mL) at 30 °C for 1 h. Subsequently, distilled water (84 mL) was added for the second hydrolysis in the autoclave at 121 °C for 1 h. The mixture was then filtered by porcelain filter crucibles with glass filters. The glucose, xylose, arabinose, mannose, galactose and rhamnose concentrations in the filtrates were determined by HPLC using a Shodex sugar SP0810 column. The cellulose content was calculated using a correction of 0.90 for glucose, and the hemicellulose content was calculated using a correction of 0.88 for the sum of xylose and arabinose content plus a correction of 0.90 for mannose, galactose and rhamnose. Lignin content (total lignin) was defined as a sum of Klason lignin residue and acid soluble lignin, and measured following the method described by Jurak, *et al*.^15^. All treatments were performed in triplicate and the test data were statistically analyzed using SPSS 19.0.

### Assay for lignocellulosic enzyme activities

Lignocellulosic enzyme activities of every composting and mushroom cultivation phases were determined including filter paper cellulase (FPUase, total cellulase activity), carboxymethyl cellulase (CMCase, endoglucanase, endo-1, 4-*β*-D-glucanase, EC 3.2.1.4), endo-xylanase (endo-1, 4-*β*-xylanase, E.C.3.2.1.8), mannanase (mannan endo-1, 4-*β*-mannosidase, EC 3.2.1.78), laccase (*p*-diphenol:dioxygen oxidoreductase, EC 1.10.3.2), and manganese peroxidase (MnP, EC 1.11.1.13). Freeze dried and milled (<1 mm) sample powders were dissolved in physiological saline (1:10, g/v), and incubated in shake flasks at 25 °C, 220 rpm for 2 h. Subsequently, the suspensions were centrifuged at 4 °C, 12000 rpm for 10 min. The supernatant was collected as the crude enzyme extract and further assayed or stored at −80 °C.

FPUase and CMCase activities were assayed using filter paper (Whatman No. 1) and CMC (Sigma-Aldrich) as substrates, respectively. Filter paper (50 mg) or 1% CMC (0.5 mL) and Na-citrate buffer (1.5 mL, 50 mM, pH 4.8) were added to a test tube. Subsequently enzyme solution (0.5 mL) was added and thoroughly mixed, followed by a constant temperature bath at 50 °C for 30 min. After incubation, the released reducing glucose was determined following the 3,5-dinitrosalicylic acid reagent (DNS) method. In brief, DNS (1.5 mL) was added into the reaction tube, followed by boiling for 5 min and cooled to room temperature. Finally, after the colored solution was diluted with 20 ml of H_2_O, the absorbance at 540 nm was measured^63–65^. The endo-xylanase^65,66^ and mannanase^67,68^ activities were measured following the same method of CMCase using xylan (Sigma-Aldrich) and locust bean gum (Sigma-Aldrich) as substrates, respectively. Standard curves of absorbance at 540 nm *vs* reducing sugar (glucose, xylose, or mannose) content were made. One enzyme unit (U) was defined as the amount of enzyme required to releasing 1 μM of reducing sugar per minute under the assay conditions^65^. All determinations were performed in triplicate. Heat-inactivated enzyme solution was used as negative control.

Laccase activity was determined using 2,2′-azino-bis (3-ethylbencentiazolin-6- sulphonic acid) (ABTS, Sigma-Aldrich) as the substrate^69,70^. Enzyme solution (10 μL) was mixed with 0.6 mM ABTS solution (290 μL, in 50 mM sodium acetate buffer, pH 5.2) at 37 °C for 5 min, followed by an addition of 5% trichloroacetic acid (TCA, 700 μL) to end the reaction. The increase in absorbance was monitored at 420 nm to test enzyme activity. MnP activity was determined using 2,6-dimethoxyphenol (DMP) as the substrate with the reaction solution of enzyme solution (100 μL), sodium tartarate buffer (690 μL, 100 mM, pH 4.5), MnSO_4_ solution (100 μL, 10 mM), H_2_O_2_ solution (10 μL, 10 mM), and DMP solution (100 μL, 10 mM). After incubation at 30 °C for 5 min, the reaction was ended by 5% TCA (500 μL) and absorbance at 420 nm was determined^43,71^. One enzyme unit (U) of laccase and MnP was defined as the amount of enzyme that oxidized 1 μmol of ABTS and DMP per minute, respectively.

### Scanning electron microscope analysis of millet straw

Fresh millet straw segments of different phases were randomly collected, fixated, dehydrated, replaced, dried, and covered with gold in an Emscope sputter coater (HITACHI E-1010, JPN). Subsequently, samples were examined using a scanning electron microscope (TESCAN TS 5136MM, CZE)^32,46^.

### 16S rRNA gene sequencing and analysis

The extraction of total genomic DNA of samples from P0, PI, and PII were carried out by using the Power Soil DNA extraction kit (MoBio Laboratories Inc., Carslab, USA)^72^. The bacterial 16S rRNA gene region V3-V4 was amplified with the primer set 338F (5′-ACTCCTACGGGAGGCAGCAG-3′) and 806 R (5′-GGACTACHVGGGTWTCTAAT-3′)^73,74^. All PCR reactions were carried out in 20 μL with 0.8 μL forward and reverse primers (5 μM), 10 ng template, 4 μL buffer (5×), 2 μL dNTPs (2.5 mM), 0.4 μL polymerase, 0.2 μL BSA. The PCR products were quantified and then constructed DNA library which was sequenced using the Illumina MiSeq PE300 platform (Illumina, San Diego, CA, USA). Raw reads were abundance filtered by removing read length lower than 50 bp, Phred score lower than 20, or containing N bases^75^. Subsequently, clean reads were clustered with the Ribosomal Database Project (RDP) Bayesian Classifier and clustered into operational taxonomic units (OTUs) at 97% identity with consensus taxonomy by Quantitative Insights into Microbial Ecology software (QIIME version 1.6.0)^73,76^. Distance based redundancy analysis (db-RDA) on Euclidean distances was performed to estimate variability in the bacterial community structure explained by physical-chemical properties^77^. The Heatmap and dbRDA were computed using the Vegan package in R^78^.

## Supplementary information

Dataset 1
