# Supplementary material for: Lignocellulose utilization and bacterial communities of millet straw based mushroom (Agaricus bisporus) production
Source: Sci Rep. 2019 Feb 4;9:1151. doi: 10.1038/s41598-018-37681-6 (PMC6362146; doi:10.1038/s41598-018-37681-6)
Supplement: Supplementary file 1 — Dataset 1 [file 41598_2018_37681_MOESM1_ESM.doc]

**Lignocellulose utilization and bacteria communities of millet straw based mushroom (*Agaricus bisporus*) production**

Hao-Lin Zhang^a1, b^, Jin-Kang Wei^c^, Qing-Hui Wang^d^, Rui Yang^a1^, Xiao-Jing Gao^a1^, Yu-Xi Sang^a1^, Pan-Pan Cai^a1^, Guo-Qing Zhang^a2,*^, Qing-Jun Chen^a1,*^

^a^ Beijing University of Agriculture, Beijing 102206, China

^1^ Beijing Key Laboratory for Agricultural Application and New Technique, College of Plant Science and Technology

^2^ Key Laboratory of Urban Agriculture (North) of Ministry of Agriculture, College of Biological Science and Engineering

^b^ College of Forestry and Horticulture, Xinjiang Agricultural University, Urumqi 830052, China

^c^ Beijing Agricultural Technology Extension Station, Beijing, 100029, China

^d^ Chengde Xingchunhe Agricultural Co. Ltd., Chengde 067000, China.

^*^ Corresponding author.

E-mail: zhanggqbua@163.com (Guo-Qing Zhang)

cqj3305@126.com (Qing-Jun Chen)

Tel & Fax: +8610-8079-7308 (Guo-Qing Zhang)

+8610-8079-9143 (Qing-Jun Chen)

**Additional file 1: Table S1 The carbohydrate compos**ition of raw straw and compost based on dry matter.

| Samples | | Glucose (%) | Xylose (%) | Arabinose (%) | Galactose (%) | Mannose (%) | Rhamnose (%) |
| --- | --- | --- | --- | --- | --- | --- | --- |
| Millet straw | | 40.75 ± 0.44 | 15.13 ± 0.06 | 2.51 ± 0.04 | 0.93 ± 0.06 | <0.01 | 1.05 ± 0.05 |
| Wheat straw-XJ | | 40.39 ± 0.75 | 17.87 ± 0.20 | 2.57 ± 0.06 | 0.71 ± 0.03 | 0.38 ± 0.09 | 1.11 ± 0.09 |
| Wheat straw-JX | | 37.75 ± 0.95 | 17.32 ± 0.86 | 2.37 ± 0.05 | 0.90 ± 0.06 | <0.01 | 1.08 ± 0.09 |
| P0 | T1 | 31.57 ± 0.39 | 14.02 ± 0.61 | 2.76 ± 0.08 | 1.22 ± 0.02 | <0.01 | 0.84 ± 0.03 |
|  | T2 | 30.63 ± 1.26 | 13.46 ± 0.45 | 2.63 ± 0.12 | 1.16 ± 0.07 | <0.01 | 0.80 ± 0.06 |
|  | T3 | 30.28 ± 2.29 | 13.01 ± 0.07 | 2.62 ± 0.07 | 1.22 ± 0.08 | <0.01 | 0.76 ± 0.05 |
| PI | T1 | 32.04 ± 0.18 | 11.03 ± 0.27 | 1.70 ± 0.05 | 0.59 ± 0.05 | <0.01 | 0.71 ± 0.08 |
|  | T2 | 29.70 ± 0.28 | 9.72 ± 0.33 | 1.19 ± 0.11 | 0.58 ± 0.06 | <0.01 | 0.75 ± 0.08 |
|  | T3 | 29.00 ± 0.54 | 9.96 ± 0.11 | 1.13 ± 0.03 | 0.53 ± 0.03 | <0.01 | 0.74 ± 0.05 |
| PII | T1 | 25.29 ± 0.52 | 8.30 ± 0.22 | 0.97 ± 0.03 | 0.44 ± 0.04 | <0.01 | 0.66 ± 0.04 |
|  | T2 | 22.51 ± 0.78 | 7.49 ± 0.09 | 0.79 ± 0.01 | 0.44 ± 0.08 | <0.01 | 0.67 ± 0.01 |
|  | T3 | 22.04 ± 0.21 | 7.50 ± 0.15 | 0.82 ± 0.04 | 0.48 ± 0.04 | <0.01 | 0.66 ± 0.02 |
| Filling | T1 | 20.75 ± 0.19 | 7.10± 0.16 | 0.87 ± 0.07 | 0.42 ± 0.03 | 0.37 ± 0.10 | 0.62 ± 0.02 |
|  | T2 | 18.16 ± 0.28 | 6.24 ± 0.08 | 0.69 ± 0.03 | 0.38 ± 0.05 | 0.23 ± 0.06 | 0.60 ± 0.06 |
|  | T3 | 17.59 ± 0.02 | 5.89 ± 0.12 | 0.69 ± 0.07 | 0.43 ± 0.01 | 0.35 ± 0.02 | 0.58 ± 0.06 |
| Pinning | T1 | 20.61 ± 0.22 | 6.79 ± 0.47 | 0.73 ± 0.07 | 0.37 ± 0.04 | 0.33 ± 0.09 | 0.64 ± 0.03 |
|  | T2 | 19.23 ± 0.35 | 6.05 ± 0.17 | 0.53 ± 0.07 | 0.35 ± 0.06 | 0.31 ± 0.02 | 0.60 ± 0.09 |
|  | T3 | 19.34 ± 0.43 | 6.06 ± 0.04 | 0.63 ± 0.02 | 0.40 ± 0.04 | 0.24 ± 0.09 | 0.63 ± 0.09 |
| 1^st^ flush | T1 | 16.93 ± 0.35 | 5.02 ± 0.21 | 0.68 ± 0.03 | 0.41 ± 0.02 | 0.36 ± 0.05 | 0.55 ± 0.07 |
|  | T2 | 12.72 ± 0.24 | 4.28 ± 0.15 | 0.52 ± 0.06 | 0.34 ± 0.02 | 0.38 ± 0.04 | 0.52 ± 0.03 |
|  | T3 | 13.71 ± 0.16 | 4.39 ± 0.07 | 0.54 ± 0.05 | 0.39 ± 0.01 | 0.36 ± 0.09 | 0.59 ± 0.01 |
| 2^nd^ flush | T1 | 14.51 ± 0.27 | 5.33 ± 0.05 | 0.66 ± 0.05 | 0.44 ± 0.07 | 0.41 ± 0.06 | 0.62 ± 0.03 |
|  | T2 | 12.09 ± 0.48 | 4.42 ± 0.04 | 0.54 ± 0.03 | 0.37 ± 0.04 | 0.37 ± 0.05 | 0.56 ± 0.03 |
|  | T3 | 12.69 ± 0.24 | 4.15 ± 0.13 | 0.47 ± 0.03 | 0.42 ± 0.05 | 0.29 ± 0.03 | 0.60 ± 0.05 |
| 3^rd^ flush | T1 | 15.76 ± 0.23 | 5.51 ± 0.09 | 0.67 ± 0.10 | 0.45 ± 0.07 | 0.37 ± 0.04 | 0.72 ± 0.02 |
|  | T2 | 11.30 ± 0.19 | 3.87 ± 0.12 | 0.46 ± 0.01 | 0.33 ± 0.01 | 0.35 ± 0.03 | 0.55 ± 0.04 |
|  | T3 | 14.09 ± 0.03 | 4.65 ± 0.04 | 0.55 ± 0.05 | 0.45 ± 0.09 | 0.35 ± 0.06 | 0.66 ± 0.03 |
